# Versatile Flexible Graphene Multielectrode Arrays

**DOI:** 10.3390/bios7010001

**Published:** 2016-12-23

**Authors:** Dmitry Kireev, Silke Seyock, Mathis Ernst, Vanessa Maybeck, Bernhard Wolfrum, Andreas Offenhäusser

**Affiliations:** 1 Institute of Bioelectronics (PGI-8/ICS-8), Forschungszentrum Jülich, 52425 Jülich, Germany; d.kireev@fz-juelich.de (D.K.); s.seyock@fz-juelich.de (S.S.); m.ernst@fz-juelich.de (M.E.); v.maybeck@fz-juelich.de (V.M.); 2 Neuroelectronics, Munich School of Bioengineering, Technical University of Munich (TUM), Germany & BCCN Munich, Boltzmannstr. 11, 85748 Garching, Germany; bernhard.wolfrum@tum.de

**Keywords:** graphene, GMEA, graphene electrodes, extracellular recordings, cell-graphene interface

## Abstract

Graphene is a promising material possessing features relevant to bioelectronics applications. Graphene microelectrodes (GMEAs), which are fabricated in a dense array on a flexible polyimide substrate, were investigated in this work for their performance via electrical impedance spectroscopy. Biocompatibility and suitability of the GMEAs for extracellular recordings were tested by measuring electrical activities from acute heart tissue and cardiac muscle cells. The recordings show encouraging signal-to-noise ratios of 65 ± 15 for heart tissue recordings and 20 ± 10 for HL-1 cells. Considering the low noise and excellent robustness of the devices, the sensor arrays are suitable for diverse and biologically relevant applications.

## 1. Introduction

Performing cellular measurements while being able to record electrical cell signals over a long period of time is important for understanding many physiological processes, including the degeneration of neuronal tissue that occurs, for example, in Alzheimer’s disease. Microelectrode arrays have been shown to be able to perform such measurements over the course of several months [1]. 

A typical microelectrode array device (MEA) consists of an assembly of metallic electrodes (10–200 µm in diameter), fabricated by complementary metal–oxide–semiconductor (CMOS) technology, passivated (to work in a liquid environment), and connected to an external measurement unit [2,3]. The most common MEA materials are gold, platinum, and titanium, whose performance have been studied for decades [4,5,6,7,8]. Nonetheless, there are several problems with conventional MEAs. First, since such devices are mostly fabricated on rigid substrates (e.g., silicon, quartz, sapphire, borosilicate glass, etc.), the final devices are difficult to integrate with soft tissue [9,10,11]. Moreover, the materials are not fully suitable to perform cell measurements due to their high mechanical mismatch compared to tissue. Therefore, there is a need to fabricate chips on materials with a lower Young’s modulus such as polyimide, whose value is about 100 times smaller than that of silicon. In addition, polyimide is well suited due to its low moisture uptake in liquid environments and its high thermal and chemical stability. 

Inkjet printing technology has evolved recently to the state where the MEA elements can be easily and cost-effectively printed on different substrates, including soft polymers [12]. Nonetheless, the inkjet-printed devices are still lacking in their performance and stability when compared to microfabricated ones. Therefore, polyimide has the advantage that, though it is soft, it is still compatible with CMOS technology.

The combination of MEAs and flexible substrates has gained much attention recently, specifically when combined with carbon-based materials like carbon nanotubes (CNTs), which exhibit great performance and flexibility [13,14]. There are several ways to build devices with carbon materials. On the one hand, the combination of carbon nanomaterials (CNTs, carbon black, etc.) and soft polymers into a single component is a promising method to fabricate new materials exhibiting outstanding properties and performance [15]. On the other side, graphene can be used for this purpose. In this regard, graphene also has many promising features such as biocompatibility [16,17], intrinsic flexibility, and excellent electrical properties [18,19], allowing applications in the high-frequency regime [20,21,22]. Furthermore, the transparency of graphene provides possibilities for the development of new tools for optogenetics [23,24,25].

In this work, we report on the fabrication of flexible and robust graphene-based microelectrode arrays on a biocompatible polyimide substrate. The devices, even after severe mechanical deformation, were used for in vitro and ex vivo extracellular recordings multiple times, providing low noise and high signal-to-noise ratio recordings. 

## 2. Materials and Methods 

### 2.1. Fabrication

**Figure 1:**
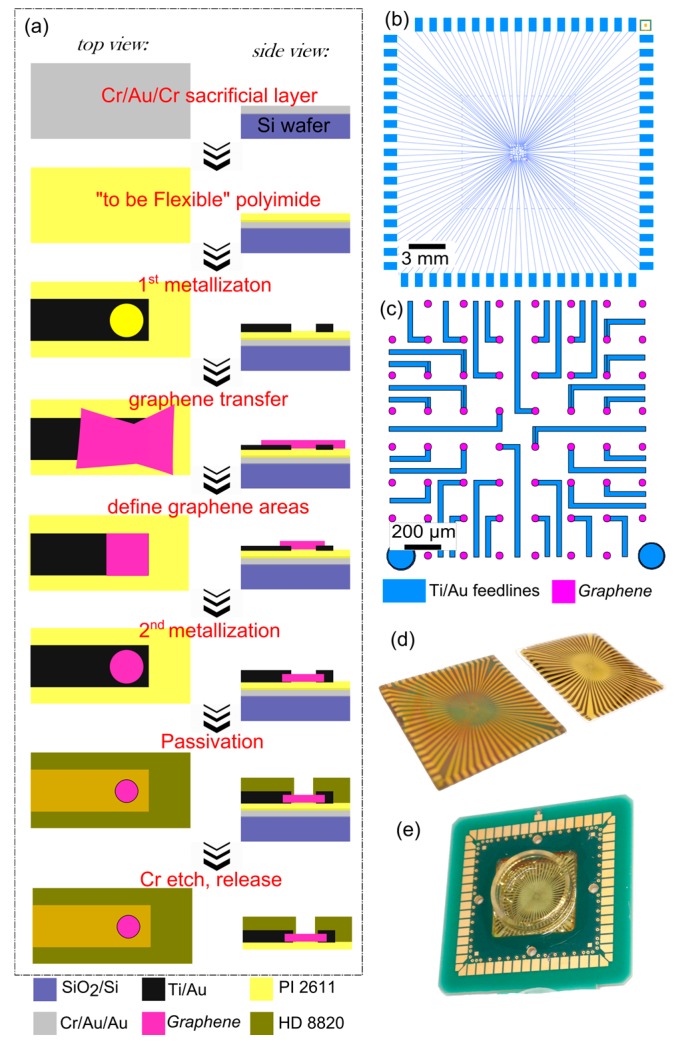
(**a**) Overview of the graphene microelectrode (GMEA) fabrication protocol; (**b**,**c**) chip design; (**d**) optical images of the chip before (left) and after (right) Cr etch; (**e**) optical image of a flexible chip, which is soldered to a carrier and encapsulated for in vitro stability.

The graphene multielectrode arrays were fabricated using standard photolithography (see Figure 1a for the fabrication steps). In order to create a flexible chip, a sacrificial layer of Cr/Au/Cr (10/100/50 nm) was evaporated on top of a Si wafer prior to the fabrication. Then, two layers of PI-2611 (HD Microsystems, Parlin, NJ, USA) were spin-coated on top of the wafer to result in an approximately 10 µm thick polyimide film after a hard-bake (350 °C). The subsequent fabrication consisted of: (1) evaporation of a metallization layer (Ti/Au, 10/50 nm) using a LOR-3B/nLOF (MicroChemicals GmbH, Ulm, Germany) resist stack for liftoff; (2) graphene transfer using a high-throughput technique [21]; (3) defining graphene areas using AZ-5214 (MicroChemicals GmbH) resist and oxygen plasma (200 sccm, 300 W, 5 min); (4) a second metallization to sandwich the graphene and provide a lower contact resistance; (5) a final passivation with photostructurable polyimide HD-8820 (HD Microsystems) resulting in an approximately 3 µm thick layer. After fabrication, the chips were immersed into chromium etchant (Sigma, St Louis, MO, USA) for approximately 24 h to remove the chromium sacrificial layer [26]. The resulting devices can be seen in Figure 1d. 

### 2.2. Soldering and Encapsulation

An important step towards stable characterization and cell culture measurements is soldering of the chips. Due to the flexibility of the devices, a standard measurement process would be difficult or even impossible to perform. A soldering protocol, as depicted in Figure S2 (Supplementary Materials) fixes the chip on a carrier and helps to improve the in vitro compatibility of the devices and the long-term stability. Special carriers were prepared, with the inner contact pads matching the exact geometry of contact pads on the chip. The carrier was placed on a hotplate (180 °C), and a soldering paste (42Sn/58Bi alloy, NC-31, AMTECH) was dispersed around the contact pads. When the flux was evaporated and the excess alloy was removed, only small amounts of alloy were left on top of the carrier’s contact pads. Afterwards, the chip was simply placed on top of the carrier and aligned under the microscope. When cooled down, any remaining flux was removed in ethanol, and the back side of the chip was glued with medical epoxy (EPO-TEK 302-3M). Two glass rings were glued (with polydimethylsiloxane (PDMS)) on top of the chip to provide containment for in vitro and ex vivo tests (see Figure S2). The suspended chip is not taut across the hole in the carrier and can still be mechanically deformed small distances. Robustness of the chips was tested by completely crumpling one of the chips prior to encapsulation (see Supplementary Materials, Video S1). Persistent wrinkles in this device further show that the encapsulated chips remain deformable after encapsulation. Therefore, the devices can be still considered to be flexible, since the substrate is not fully tensioned while soldering.

### 2.3. Electrical Impedance Spectroscopy (EIS)

Electrical impedance spectroscopy was performed on a VSP-300 multichannel potentiostat (BioLogic Science Instruments, Seyssinet-Pariset, France). The spectra were taken using graphene as a working and a Ag/AgCl pellet as a reference electrode in 1× phosphate-buffered saline (PBS) solution. A 10 mV alternating current (AC) potential was applied and a frequency range between 1 Hz and 1 MHz was scanned.

### 2.4. Multichannel Recordings

The multichannel recordings were taken using a homebuilt setup, which consists of a preamplifier (10×) and a main amplifier (1×, 10×, or 100×) [27,28,29]. The recordings can be performed at up to 64 channels in parallel with a sampling rate of 10 kHz per channel. The measurements were performed in a shielded metal box to avoid external noise sources. Only a 50 Hz comb filter was applied to the recording in order to remove the power grid pick-ups. A Ag/AgCl pellet electrode was used as a reference and connected to the ground. 

### 2.5. HL-1 Culture

The cardiomyocyte-like cells, HL-1 [30], were cultured in T25 flasks. After reaching confluency, the cells were split and seeded on top of the graphene microelectrodes (GMEAs) with a density of 200 cells/mm^2^. The details of the procedure can be found elsewhere [30]. In order to improve cellular adhesion, the chips were cleaned in 70% ethanol and coated with fibronectin/gelatin solution (5 µg/mL and 0.2 µg/mL respectively) for 1 h at room temperature. After cell seeding the chips were placed in an incubator (37 °C and 5% CO_2_). Claycomb medium, supplemented with 10% fetal bovine serum, 100 U/mL–100 µg/mL penicillin-streptomycin, 0.1 mM norepinephrine, and 2 mM l-glutamine was exchanged every day. The medium was also exchanged approximately 2 h before the measurements. 

### 2.6. Acute Heart Tissue Preparation

In order to perform ex vivo heart tissue measurements, embryonic heart tissue was prepared by dissection of an E18 Wistar rat. A heart from an embryo was quickly isolated, washed in Hank’s balanced salt solution. The following short-term storage and measurements were performed in the supplemented Claycomb medium (described above) at room temperature. The experiments were done with the approval of the Landesumweltamt für Natur, Umwelt und Verbraucherschutz Nordrhein-Westfalen, Recklinghausen, Germany, number 84-02.04.2015.A173. 

## 3. Results and Discussion

The fabricated devices are 24 × 24 mm^2^ in size (see Figure 1b), and approximately 13 µm thick with a polyimide base and polyimide passivation. The chip’s layout can be seen in Figure 1b with the feedlines aiming into the middle of the chip. The middle array of the chip (2 × 2 mm^2^ in size, see Figure 1c) is where the metal feedlines have an opening with graphene underneath (see Figure 1a). The passivating polyimide layer is developed in a way that only the graphene parts are exposed to the liquid. The final GMEAs have circular recording apertures of 20 µm in diameter. Once the devices are fabricated, the sacrificial layer is etched, releasing the flexible chips (see Figure 1d), which are further connected to a carrier for in vitro studies (see Figure S2 for a detailed description and Figure 1e for the final result). The suspended devices are constrained, but not immobilized, on the carrier. Usage of a carrier is necessary for in vitro experiments due to requirements of handling and connection to the amplifier. Yet, usage of the carrier does not fully constrain the flexibility of the devices. 

**Figure 2:**
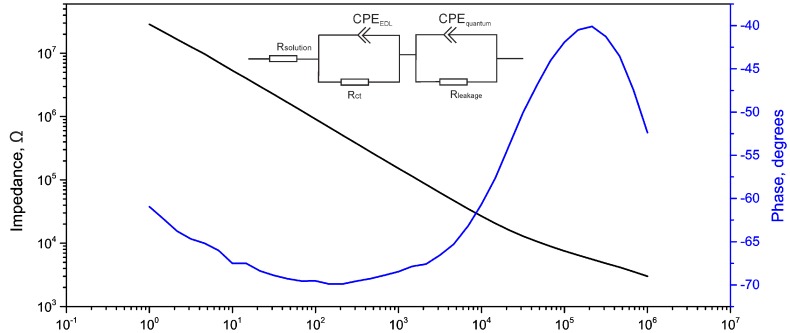
Bode plot of a GMEA with an electrode diameter of 20 µm. A linear dependency of the impedance at lower frequencies is typical of graphene electrode behavior. The inset gives the model used for fitting the data.

An electrical impedance spectroscopy Bode plot from a GMEA is given in Figure 2. Impedance of the GMEAs, of 20 µm in diameter, measured at 1 kHz is around 1 ± 0.5 × 10^5^ Ω, which is in the range of previously reported values [24,25,31,32]. In order to fit the GMEA’s behavior, one has to consider another constant phase element (compared to metal electrodes), representing quantum capacitance [33,34,35]. The general equivalent circuit used in this work is shown in the inset of Figure 2. The two constant phase elements represent the electrical double-layer and quantum capacitance. In previous works [24,31], a Warburg element was used to model linear diffusion. However, in our case, the electrode diameter is too small to be described by linear diffusion; therefore, the Warburg element was not used in our calculations. The equivalent circuit fitting values that were used are represented in Table 1 together with comparison to previously published works. 

For the recording of the cardiac cellular signal, as well as heart-tissue signal, the graphene microelectrodes function as capacitive sensing element. Assuming a point-contact model [36] of coupling between the cell and the electrode, the depolarization of the cell membrane is coupled to the graphene electrode across a small gap, where the cell or tissue adheres to the surface. The potential change is then detected by the graphene electrodes via capacitive coupling [27,28,29]. The measurement setup is explained in detail in the experimental section. 

**Figure 3:**
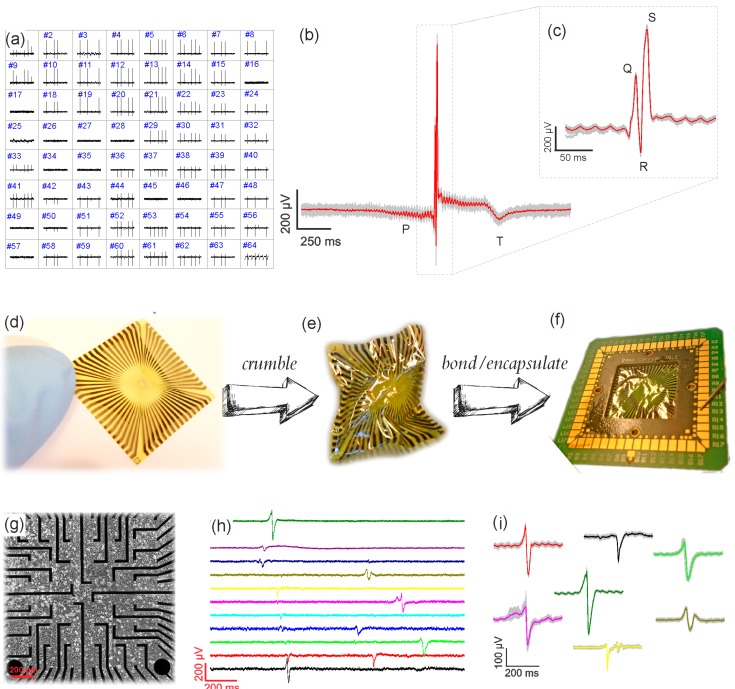
(**a**) The spatial resolution map of heart tissue recordings from a GMEA device. The distance between the electrodes is 200 µm in each direction; (**b**,**c**) the zoom-in into one action potential of 2 s and 200 ms long are given for a clear observation of P, Q, R, S, and T regions; (**d**) one flexible chip, which was crumpled (**e**), then bonded and encapsulated (**f**); (**g**) a differential interference contrast (DIC) picture of HL-1 cells grown on top of a GMEA surface; (**h**) time trace recordings of HL-1 cells from eleven channels on one GMEA chip showing a time delay in recording of different electrodes that reflects spatial propagation; (**i**) the variety of different HL-1 action potential shapes recorded with the GMEA due to differences in cell–chip coupling.

Embryonic heart tissue, extracted as described in the experimental section, was placed on top of a GMEA chip’s surface. No adhesion promoter was used, but only a small drop of a supplemented medium (30 µL) was dispersed on top and around the tissue in order to provide physiological conditions. A Ag/AgCl reference electrode was placed on top of the liquid and as close to the tissue as possible to yield a stable reference potential. The heart’s electrical activity was detected on more than 80% of all electrodes on the GMEA, as shown in the spatial diagram (see Figure 3a). The shape of the recorded potentials (see an average action potential (AP) in Figure 3b,c) clearly resembles the P, Q, R, S, and T regions of an electrocardiogram [37,38,39]. 

The average heart tissue spike amplitudes recorded here are in the range of 1 ± 0.2 mV, while noise is in the range of 20 ± 6 µV (2·MAD, mean absolute deviation) or 43 ± 11 µV (RMS, root mean square). Since the noise was calculated over a large timescale (>100 s), the presence of large amplitude spikes usually results in overestimation of the RMS value (see Supplementary Material, Figure S3); therefore, for further calculations we use 2·MAD values for noise analysis. The final signal-to-noise ratio (SNR) of the acute heart tissue recording done by the flexible GMEAs, calculated as signal amplitude divided (2·MAD), is in the range of 50 ± 15, which is well above or comparable to previous work [38].

Prior to the HL-1 cell culture, one of the GMEA chips was tested for its mechanical stability: the flexible chip was crumpled severely (see Figure 3d,e and Video S1) before soldering. In spite of the mechanical deformation, the chip has been soldered and encapsulated with the same procedure as described in the experimental section (see Figure 3f).

HL-1 cells were plated, as described in the experimental section, and incubated until confluent and contracting (usually 3 days). As a cardiac muscle cell line, they tend to form a continuous layer while growing on a surface. An optical image of such a continuous cellular layer on top of a GMEA chip is shown in Figure 3g. APs, which can be described as a change in the cellular membrane potential, are produced repeatedly, continuously, and through the whole layer. In Figure 3h, 11 traces recorded from different channels (electrodes) on the same chip are shown. The figure shows that there is a shift between the occurrences of the APs at different channels, which shows that the electrical signal propagates through the cellular layer [40]. As the signal is picked up by different electrodes with some time delay, it is evident that the spikes are not caused by noise or electronic artifacts. The AP’s amplitude, width, and shape can be different from channel to channel, but stays consistent in one channel. The main reason for different AP shapes is cell–chip coupling, which, in the case of HL-1 cells, is a more relevant parameter compared to the above-reported heart tissue signals. While growing, HL-1 cells form a continuous layer of electrically active cells connected via gap junctions [4,40,41]. Sizes of connected cell layers exceed millimeters and can be even centimeters if the monolayer is completely uniform. In such an ideal case, all electrodes might have similar coupling. Nonetheless, in most experiments the coupling varies from electrode to electrode [40,42]. Moreover, the position of the electrode completely under a cell or under the border between cells contributes to the variation in spike shape. The main waveforms of the APs are represented in Figure 3i, and the results are in accordance to the previously published works [40,43,44,45]. 

Lastly, signal-to-noise ratios were calculated for the HL-1 recordings. Since the cardiac cells’ recorded spike amplitudes typically did not exceeding 300 µV, the overall SNR for such recordings is 20 ± 10. The SNR is in same range as the SNR of recordings from state-of-the-art microelectrode configurations (see Supplementary Materials, Table S1). 

## 4. Conclusions 

The presented GMEA devices show extracellular recordings with excellent signal-to-noise ratios of up to 65 ± 15. The use of graphene’s extraordinary properties for fabrication of electrode arrays on a biocompatible polyimide substrate results in good cell-interface properties and is promising for further applications. Due to the transparency of our devices, the concept can be extended for optogenetic experiments. Furthermore, the fabrication technique explored in the manuscript can be adjusted for the design of in vivo devices such as bioimplants.

## Figures and Tables

**Table 1:** Fitting results for electrical impedance spectroscopy (EIS) measurements and their comparison to previously published works.

	Used Model	R_S_ (kΩ)	C_PE1_ (S·s^n^)		R_CT_ (Ω)	Z_W_ (S·s^1/2^)	C_PE2_ (S·s^n^)		R_L_ (Ω)	Area
			Q	n			Q	n		
This work	R_S_ + Q_2_/R_2_ + Q_3_/R_3_	0.8	5.0 × 10^−9^	0.68	5.8k	–	7.36 × 10^−9^	0.78	140M	314 µm^2^
Kuzum et al. [24]	R_S_ + Q_2_/(R_2_ + W_2_)	–	5.6 × 10^−9^	0.67	85M	17.36 × 10^−9^	–	–	–	2500 µm^2^
Du et al. [31]	R_S_ + Q_2_/(R_2_ + W_2_) + Q_3_/R_3_	0.17	5.75 × 10^−7^	0.67	3k	8.12 × 10^−6^	5.55 × 10^−7^	0.9	23.4M	7000 µm^2^

## Supplementary Materials

The following are available online at www.mdpi.com/2079-6374/7/1/1/s1, Table S1: Comparison of the SNR of the extracellular recording with different kinds of MEAs; Figure S1: Raman spectra of used grapheme; Figure S2: soldering flexible chip: overview; Figure S3: RMS vs. MAD noise comparison; Video S1: crumbling the flexible chip.
