# Supplementary material for: Versatile Flexible Graphene Multielectrode Arrays
Source: Biosensors (Basel). 2016 Dec 23;7(1):1. doi: 10.3390/bios7010001 (PMC5371774; doi:10.3390/bios7010001)
Supplement: Supplementary file 1 [file biosensors-07-00001-s001.pdf]

# Supplementary Materials: Versatile Flexible Graphene Multielectrode Arrays

**Dmitry Kireev, Silke Seyock, Mathis Ernst, Vanessa Maybeck, Bernhard Wolfrum and Andreas Offenhäusser**

**Table S1.** Comparison of the signal-to-noise ratio (SNR) of the extracellular recording with different kinds of microelectrode arrays (MEAs).

| | Active Material | Substrate | Electrode Diameter | Tissue Type | SNR |
|---|---|---|---|---|---|
| This work | Graphene | Polyimide | 20 µm | Heart tissue<br>HL-1 | 65 ± 15 [(a)]<br>20 ± 10[(a)] |
| Du et al. [1] | Graphene | Polyimide | 20 µm | Neurons | 10.3 ± 1.2 |
| Brüggemann et al. [2] | Au planar | SiO2/Si | 10 µm<br>20 µm | HL-1 | 57 [(b)]<br>141 [(b)] |
| Hofmann et al. [3] | Au nanocavity | SiO2/Si | 10 µm | HL-1 | 158 ± 8 |
| Nick et al. [4] | Au<br>Rough TiN | Quartz | 30 µm | Chicken cardiomyocytes | 10 [(c)]<br>207 [(c)] |
| James et al. [5] | Au planar<br>Pt nonplanar | Fused silica | 12 µm | Neurons | 15 ± 10 |

(a) Noise defined as 2*MAD (mean absolute deviation); (b) Noise defined as root mean square (RMS); (c) SNR defined as variance $((v_{peak}/\sigma_{noise})^2)$.

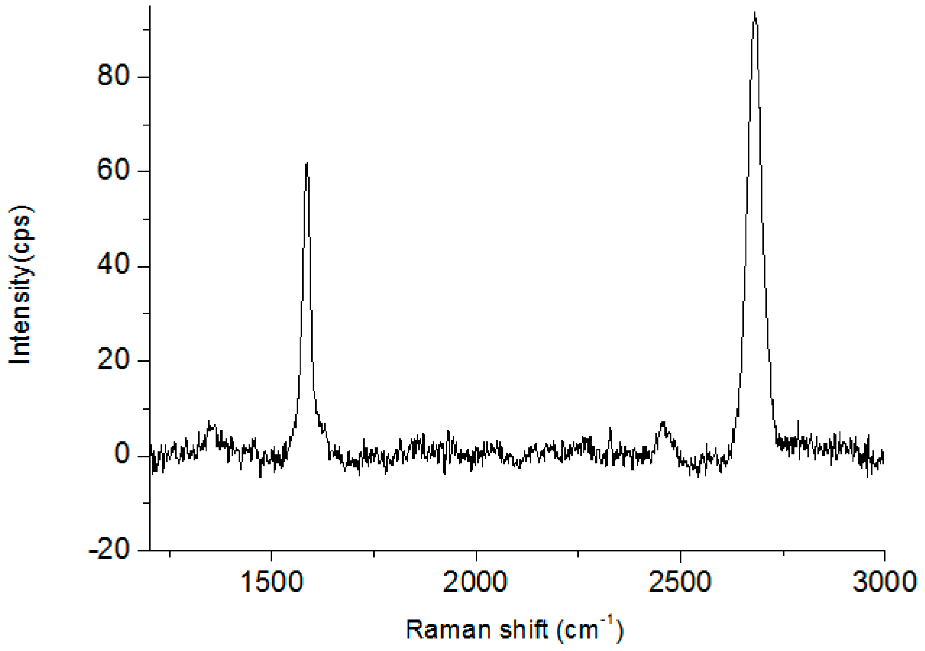


**Figure S1.** Raman spectra of the chemical vapor deposition (CVD)-grown graphene used. Almost-absent D peak shows that the graphene is without defects.

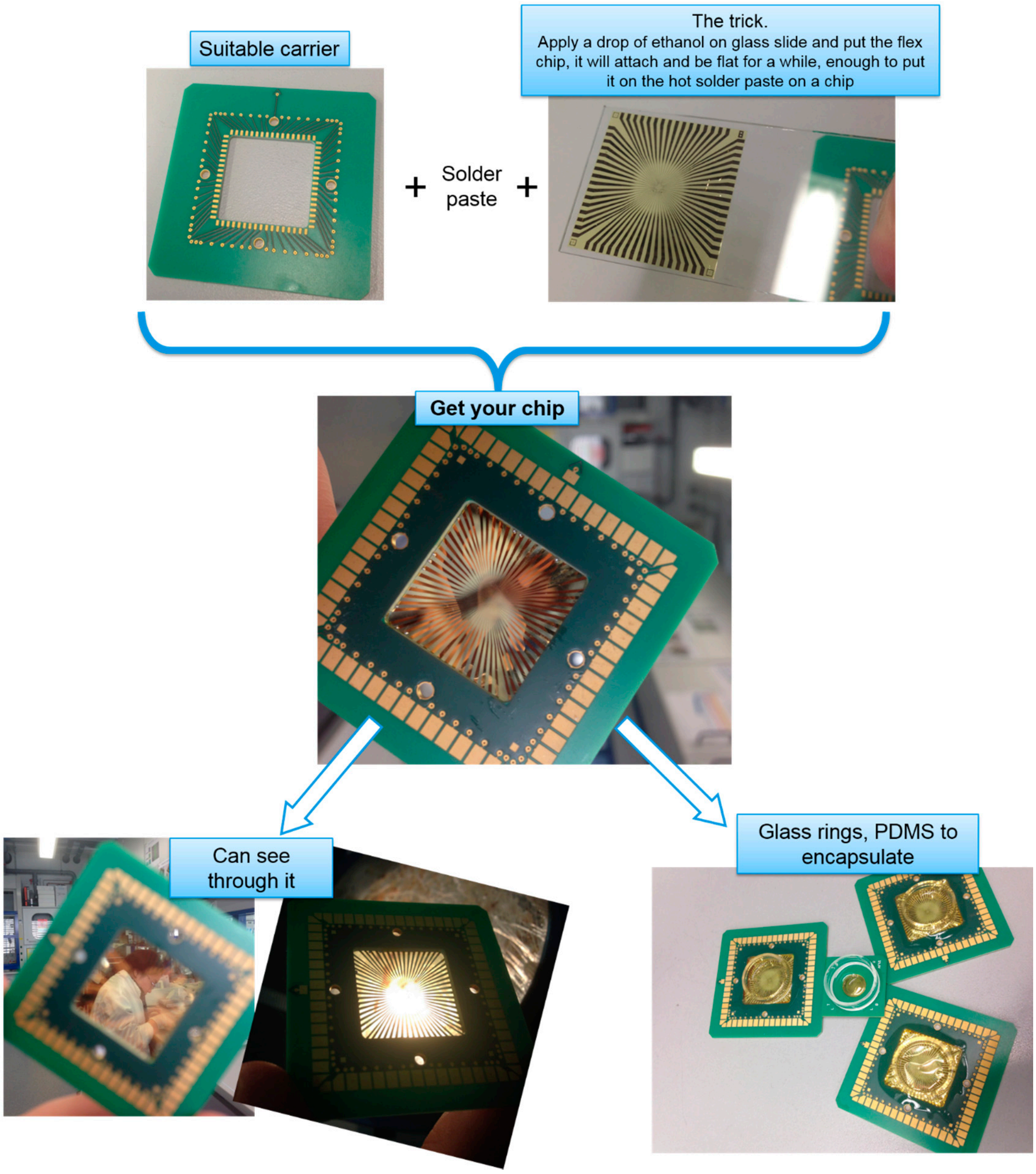


**Figure S2.** Soldering flexible chip: overview.

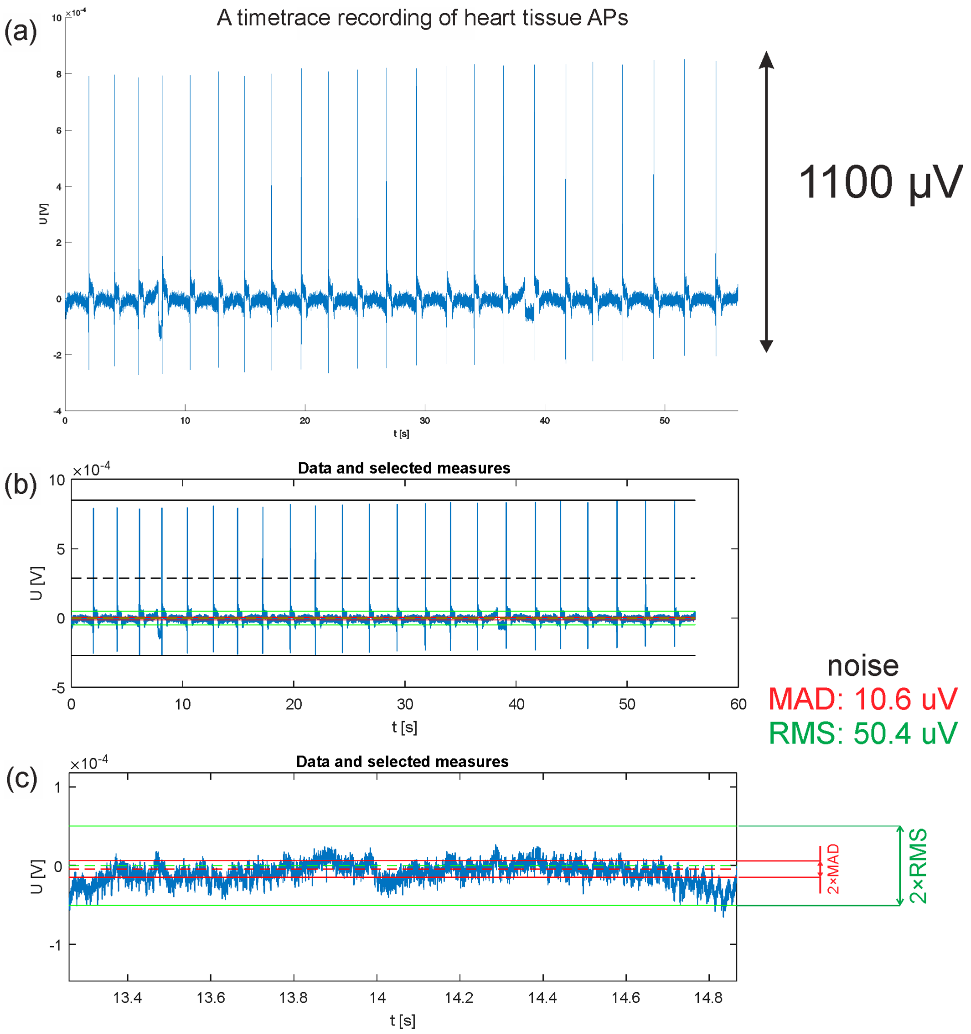


**Figure S3.** MAD vs RMS noise value comparison. For a long recording, the noise is usually evaluated for the whole time (**a**,**b**). In this case, the RMS value will be overestimated, while the 2*MAD value better approximates the peak-to-peak noise in the interspike interval, as can be seen in (**c**).

## References


1. Du, X.; Wu, L.; Cheng, J.; Huang, S.; Cai, Q.; Jin, Q.; Zhao, J. Graphene microelectrode arrays for neural activity detection. *J. Biol. Phys.* **2015**, *41*, 339–347.
2. Brüggemann, D.; Wolfrum, B.; Maybeck, V.; Mourzina, Y.; Jansen, M.; Offenhäusser, A. Nanostructured gold microelectrodes for extracellular recording from electrogenic cells. *Nanotechnology* **2011**, *22*, 265104.
3. Hofmann, B.; Kätelhön, E.; Schottdorf, M.; Offenhäusser, A.; Wolfrum, B. Nanocavity electrode array for recording from electrogenic cells. *Lab Chip* **2011**, *11*, 1054–1058.
4. Nick, C.; Joshi, R.; Schneider, J.J.; Thielemann, C. Three-dimensional carbon nanotube electrodes for extracellular recording of cardiac myocytes *Biointerphases* **2012**, *7*, 1–6.
5. James, C.D.; Spence, A.J.H.; Dowell-Mesfin, N.M.; Hussain, R.J.; Smith, K.L.; Craighead, H.G.; Isaacson, M.S.; Shain, W. Turner, J.N. Extracellular recordings from patterned neuronal networks using planar microelectrode arrays *IEEE Trans. Biomed. Eng.* **2004**, *51*, 1640–1648.
